# Injectable, self-healing mesoporous silica nanocomposite hydrogels with improved mechanical properties†

**DOI:** 10.1039/d0nr07406c

**Published:** 2020-12-22

**Authors:** A. Zengin, J. P. O. Castro, P. Habibovic, S. H. van Rijt

**Affiliations:** Department of Instructive Biomaterials Engineering (IBE), MERLN Institute for Technology-Inspired Regenerative Medicine, Maastricht University the Netherlands s.vanrijt@maastrichtuniversity.nl

## Abstract

Self-healing hydrogels have emerged as promising biomaterials in regenerative medicine applications. However, an ongoing challenge is to create hydrogels that combine rapid self-healing with high mechanical strength to make them applicable to a wider range of organs/tissues. Incorporating nanoparticles within hydrogels is a popular strategy to improve the mechanical properties as well as to provide additional functionalities such as stimuli responsiveness or controlled drug delivery, further optimizing their use. In this context, mesoporous silica nanoparticles (MSNs) are promising candidates as they are bioactive, improve mechanical properties, and can controllably release various types of cargo. While commonly nanoparticles are added to hydrogels as filler component, in the current study we developed thiol surface-functionalized MSNs capable of acting as chemical crosslinkers with a known hydrophilic polymer, polyethylene glycol (PEG), through dynamic thiol–disulfide covalent interactions. Due to these dynamic exchange reactions, mechanically strong nanocomposites with a storage modulus of up to 32 ± 5 kPa compared to 1.3 ± 0.3 kPa for PEG hydrogels alone, with rapid self-healing capabilities, could be formed. When non-surface modified MSNs were used, the increase in storage modulus of the hydrogels was significantly lower (3.4 ± 0.7 kPa). In addition, the nanocomposites were shown to degrade slowly over 6 weeks upon exposure to glutathione while remaining intact at physiological conditions. Together, the data argue that creating nanocomposites using MSNs as dynamic crosslinkers is a promising strategy to confer mechanical strength and rapid self-healing capabilities to hydrogels. This approach offers new possibilities for creating multifunctional self-healing biomaterials for a wider range of applications in regenerative medicine.

## Abstract 2

Nanocomposites created by dynamic crosslinking of mesoporous silica nanoparticles to PEG hydrogels represents a promising strategy to confer mechanical strength and rapid self-healing capabilities to hydrogels.

## Graphical abstract

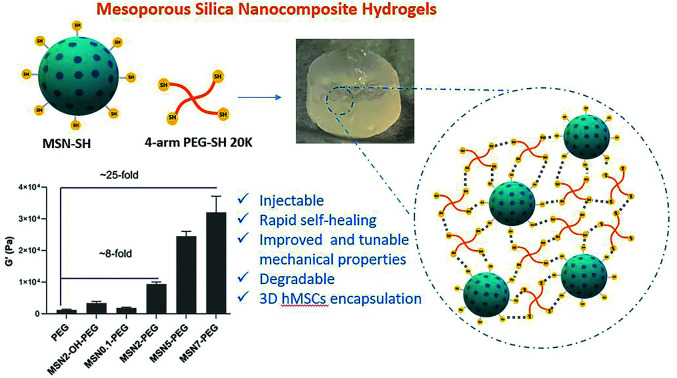

## 1. Introduction

Hydrogels are three-dimensional (3D) polymeric networks that can absorb high amounts of water, and have tunable chemical, physical, and biological properties. These features make them ideal materials to mimic the extracellular matrix of native tissue.^1^ As such, hydrogels are a promising class of soft materials for many biomedical applications including tissue engineering, drug delivery, and wound healing.^2^ More recently, inspired by the autonomous healing process of natural tissues and given their stability and durability, self-healing hydrogels have received great attention to replace the often brittle hydrogels currently investigated in clinical applications.^3–5^ In particular in tissue engineering applications, the self-healing properties together with injectability allow such materials to reach deep into tissue, and the possibility to adapt to fill irregular geometries in damaged areas in a minimally invasive manner.^6,7^ Strategies to design injectable, self-healing hydrogel networks include the use of reversible non-covalent interactions^3–5,7^ (*e.g.* hydrogen bonds, ionic bonds, host–guest interactions) and dynamic covalent interactions^8–11^ (*e.g.* Diels–Alder reactions, acyl hydrazone bonds, and disulfide bonds). Among dynamic covalent interactions, a thiol–disulfide exchange reaction is an attractive approach since it takes place under ambient conditions without external factors such as high temperature^10^ (as is the case with Diels–Alder reactions) or acidic environments^9^ (in hydrazone bonds). During these reactions, thiol radicals are formed through the breakage of disulfide bonds, which can readily attack other disulfide bonds to form new covalent bonds.^8,11^ Moreover, disulfide bonds can introduce stimuli responsiveness to the hydrogel matrix because they can be degraded in the presence of glutathione, a reducing agent found in the body.^8^

Despite these efforts in the design and development of injectable, self-healing hydrogels, most are not able to adapt to the dynamic and mechanically demanding environment of (stiffer) organs and tissues. As such, an ongoing challenge is to combine high material strength with a fast self-healing ability. To date, different strategies have been used to improve the mechanical properties of hydrogels, where increasing polymer concentration or the number of crosslinking bonds within the network has received most attention.^12–15^ Another approach incorporates nanoparticles into the hydrogel network to improve the mechanical properties and bioactivity of the hydrogel matrix. Various types of nanoparticles, including polymeric, gold, and silicate, have been employed to generate nanocomposites for biomedical applications.^16,17^ Incorporation of nanoparticles provides additional features to create responsive nanocomposites that contain *e.g.* electric, magnetic, conductive or improved bioactivity.^18–21^

Mesoporous silica nanoparticles (MSNs) are interesting nanomaterials due to their biocompatibility, high surface area, stable and homogeneous structure, easy modification of core and shell chemistry, and porous structure.^22,23^ Hydrogels incorporating MSNs have shown increased bioactivity and mechanical properties.^15,24–32^ For example, Liu *et al.* demonstrated that the Young's modulus of PEGDA hydrogels could increase 3-fold in the presence of 10 wt% MSNs within the gel network.^15^ Similarly, Gaharwar *et al.* reported that MSNs could increase mechanical strength and toughness of photocrosslinked PEG hydrogels and enhance adhesion, spreading, and metabolic activity of fibroblasts.^26^ In a different study, Alonci *et al.* developed *in situ*-forming, injectable nanocomposite hydrogels based on covalent bond breakable MSNs and polyamidoamines as a potential submucosal injection agent for use in endoscopic submucosal dissections. The resulting nanocomposite hydrogels were biocompatible and degraded completely in response to cell-secreted glutathione.^33,34^ Furthermore, the pores of the MSNs have been employed as reservoirs for small molecules such as drugs or peptides to improve the bioactivity of the nanocomposites.^35,36^ For example, Zhu *et al.* developed an MSN–chitosan nanocomposite for sustained co-delivery of small chemotherapeutic drugs and biomacromolecules. Two model drugs, gentamicin and bovine serum albumin, could be delivered in a sustained manner from the MSN–chitosan hydrogels in 7 days.^32^

**Scheme 1:**
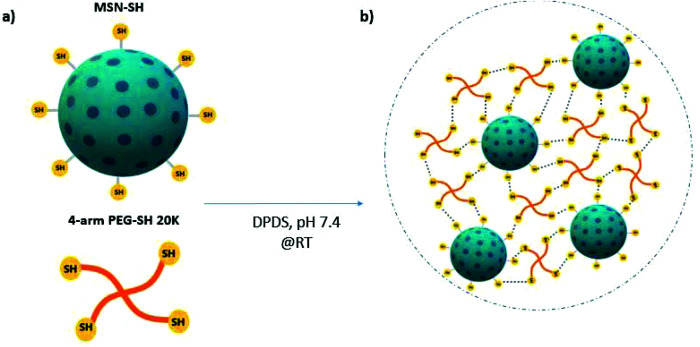
Formation of MSN-PEG nanocomposite hydrogels *via* dynamic covalent interactions. (a) Chemical structure of MSN-SH and 4-arm-PEG-SH. (b) Hydrogel formation through the thiol–disulfide exchange reactions at room temperature.

In the above mentioned and most other reported nanocomposite materials, the nanoparticles are introduced as filler components or as physical crosslinkers within the polymeric matrix. In such materials, the nanoparticles do not actively take part in the hydrogel network formation. Herein, we developed thiol surface-functionalized MSNs (MSN-SH) that are capable of acting as chemical crosslinkers with thiol-modified polyethylene glycol (PEG-SH) through dynamic covalent interactions. We postulated that mechanically strong nanocomposites with rapid self-healing capabilities can be formed due to thiol–disulfide exchange reactions between MSN-SH and 4-arm-PEG-SH (Scheme 1). PEG was selected as a model synthetic polymeric backbone due to its well-known biocompatibility (FDA approved), and tunable structural properties.^37^ We investigated the mechanical and self-healing properties of these 3D networks by oscillatory rheology. Furthermore, the mass loss method was used to analyze their degradation behavior in the presence of a glutathione-containing environment. We also calculated the equilibrium swelling degree of the hydrogels and tested their drug-release properties by encapsulating rhodamine B and albumin-FITC. Finally, cytocompatibility of these networks was evaluated by staining hMSCs-encapsulated hydrogels using phalloidin and DAPI.

## 2. Material and methods

### 2.1 Materials

Tri-ethanolamine (TEA), tetraethyl orthosilicate (TEOS), 3-aminopropyl triethoxysilane (APTES), triethoxyphensylsilane (PTES), 3-mercaptopropyl triethylsilane (MPTES), cetyltrimethylammonium chloride (CTAC), absolute ethanol, hydrochloric acid (HCl, 37%), ammonium nitrate (NH_4_NO_3_), 5/6-carboxyfluorescein succinimidyl ester (NHS-FITC) and ATTO 633-maleimide. 2,2′-Dipyridyldisulfide (DPDS), 4arm-PEG20K-SH, rhodamine B (RhoB), glutathione reductase (GSH) were purchased from Sigma Aldrich GmbH (Germany). Absolute ethanol, paraformaldehyde (PFA), Triton X-100, bovine serum albumin (BSA), Tween-20 were purchased from VWR (US).

### 2.2 MSN synthesis

MSNs with amine functional groups in the core and thiol functional groups on the surface were synthesized as follows: a mixture of TEA (14.3 g, 95.6 mmol), TEOS (1.466 g, 7.37 mmol), PTES (110.8 mg, 0.461 mmol), and APTES (82.65 mg, 0.461 mmol) was heated at 90 °C for 20 min without stirring (Solution 1). A solution of ammonium fluoride (100 mg, 2.7 mmol), CTAC (2.41 ml, 1.83 mmol) in 21.7 ml mili Q-water (Solution 2) was heated at 60 °C for 20 min. Solution 2 was rapidly added to solution 1, and the mixture was left stirring at 700 rpm for 20 min while cooling down to room temperature (RT). Next, TEOS (138.2 mg, 0.922 mmol) was added to the solution mixture in four equal increments (approx 37 μl) every 3 min, and the mixture was left stirring for another 30 min at RT. Later, TEOS (19.3 mg, 92.5 μmol) and MPTES (20.5 mg, 92.5 μmol) were added to the mixture and left stirring overnight at RT. The particles were collected by centrifugation at 7500 rpm for 20 min and washed once with absolute ethanol. For organic template extraction, the particle suspension was heated for 45 min under reflux (90 °C) in an acidic ethanol solution (2 g NH_4_NO_3_ in 100 ml ethanol). MSNs were collected by centrifugation and washed with absolute ethanol before second template extraction in 100 ml of a 3.7% hydrochloric acid solution in ethanol for 45 min at 90 °C. After two washing steps with absolute ethanol, the MSN suspension was stored at −20 °C in absolute ethanol.

### 2.3 Characterization of MSNs

The hydrodynamic diameter and zeta potential of synthesized MSNs (0.5 mg mL^−1^) were determined by dynamic light scattering using a Malvern Zetasizer Nano (Panalytical, UK). The shape and porosity of the nanoparticles were imaged using scanning electron microscopy (SEM; Teneo, FEI, US) and transmission electron microscopy (TEM; FEI Tecnai electron microscope). For SEM imaging, the samples were sputter-coated with 4 nm iridium. For TEM imaging, MSN suspension in ethanol was dropped on a TEM carbon grid and imaged after air drying at RT.

To confirm the successful core–shell functionalization, MSNs were labeled with fluorescent dyes. 5/6-Carboxyfluorescein succinimidyl ester (NHS-FITC) and ATTO 667N-maleimide were used to label the amine groups in the core and the thiol groups on the surface, respectively. Per reaction, 20 μL of the FITC-NHS solution (6.3 mg mL^−1^ in absolute ethanol) and 0.5 μL of ATTO dye solution (5 mg mL^−1^ in DMF) were used to label per 1 mg of MSN. Coupling reactions of the dyes with MSNs were performed in ethanol during overnight stirring. MSNs were collected by centrifugation (14 000 rpm, 20 min) and washed three times with absolute ethanol. MSNs were dispersed in absolute ethanol at a concentration of 10 mg mL^−1^ and fluorescent measurements were performed using a ClARIOstar spectrophotometer (BMG LABTECH, Germany). The fluorescent signal for FITC labeling was detected at *λ*_Ex_ = 488–14 nm and *λ*_em_ = 535–30 nm and for ATTO at *λ*_ex_ = 620–30 nm and *λ*_em_ = 680–40 nm.

### 2.4 Hydrogel formation

Nanocomposite hydrogels were formed through the mixture of freeze-dried MSN-SH and 4-arm-PEG-SH in PBS (2 wt% and 10 wt%, respectively) in presence of 2,2′-dipyridyl disulfide (DPDS, 8 mM in PBS–ethanol solution) as an initiator. The volumetric ratio between the MSN-PEG solution and DPDS was 3 : 1. Hydrogels were left to equilibrate at RT for 30 min. The control hydrogels based on 4-arm-PEG-SH was formed upon mixing PEG solution (10 wt% in PBS) with DPDS solution in a 3 : 1 ratio.

### 2.5 Rheological characterization of hydrogels

The mechanical properties of the hydrogels were determined by performing time sweeps, frequency sweeps, strain sweeps using oscillatory rheology (TA instruments, Discovery HR-2). Gelation kinetics of the hydrogels was observed by oscillatory time sweep test at a constant frequency (*ω*) of 1 Hz and a 1% strain amplitude (*γ*). The frequency sweep test was carried out on the hydrogels at a constant strain of 1% and covered a range of frequencies from 0.1 to 10 Hz. Finally, a strain sweep was performed from 0.1% to 1000% strain at a constant frequency (1 Hz). The self-healing behavior was studied by a cyclic strain sweep. In these measurements, the strain amplitude was shifted between 500% and 1% for four cycles at a constant frequency of 1 Hz. All the measurements were done using a 20 mm (diameter), 2° cone geometry with a gap of 53 μm at 20 °C and analyzed using TA Instruments TRIOS software. The hydrogels were formed *in situ* on the rheometer stage by mixing 60 μl of MSN-PEG solution and 20 μl of DPDS solution in the center of the stage.

### 2.6 Surface analysis of hydrogels

SEM imaging (Teneo, FEI, US) was used to analyze the surface properties of the hydrogels. After hydrogels were formed and left to equilibrate at room temperature for 30 min, they were frozen at −20 °C and then lyophilized overnight. Before SEM imaging, the hydrogels were sputter-coated with 4 nm iridium.

### 2.7 Equilibrium swelling and degradation behavior of the MSN-PEG hydrogels

Equilibrium swelling behavior of the hydrogels was studied by comparing the mass of the hydrogels directly after gelation with the mass of the hydrogels after swelling. First, hydrogels were prepared in Eppendorf tubes of which the weights (*W*_E_) were recorded before experiments, in a total volume of 100 μl and left to incubate for 30 min at RT to reach an equilibrium. Afterward, the hydrogels were weighed on an analytical balance (*W*_0_), and 400 μl of PBS solution was added on top of the hydrogels. At predetermined time intervals, the PBS solution on top of the hydrogels was removed and the swollen weights of the hydrogels were measured (*W*_t_). Fresh PBS solution was put into tubes after recording their weight. The equilibrium swelling ratio degree of hydrogels was calculated by the following equation:1
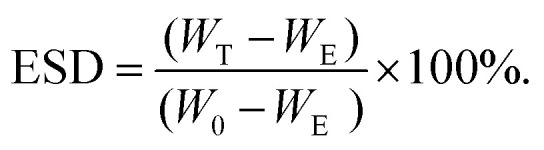


The degradation behavior of the hydrogels was examined by using the mass loss method in the presence of glutathione (GSH), which reduces the disulfide bonds. For these studies, mild (300 μm) and excessive glutathione (10 mM) conditions were chosen. Similar to swelling experiments, the weights of empty Eppendorf tubes (*W*_E_) were recorded before experiments. Hydrogels were prepared in these tubes and left to equilibrate for 30 min. Then, phosphate-buffered saline (PBS) was added on top of the hydrogels and they were left to incubate at 37 °C for 24 h. Next, PBS media was removed and the mass of the tubes (*W*_0_) was weighted. Afterward, swelling media (300 μM glutathione or PBS as control) was added on top of the hydrogels and the tubes were left to incubate at 37 °C. At predetermined time intervals, the swelling media was removed and swollen weights of hydrogels (*W*_t_) were measured on an analytical balance. The weight remaining ratio of hydrogels was calculated by using eqn (1).

### 2.8 Bulk release of a model dye from the nanocomposite hydrogels

RhoB and Albumin-FITC were used as the model drug to analyze the drug release characteristic of the hydrogels. Both dyes were encapsulated inside the hydrogel network during the gel formation. First, lyophilized MSN-SH (2 wt% and 7 wt%) and 4-arm-PEG-SH (10 wt%) were mixed in PBS at pH 7.4 (Solution 1). Then, RhoB and Albumin-FITC were dissolved in separate DPDS solutions to reach a concentration of 500 μM and 2.5 mg ml^−1^, respectively (Solution 2). Then, 150 μl of solution 1 was mixed with 50 μl of solution 2 in disposable UV-cuvettes and left to incubate for 1 h at RT. Next, 2300 μl PBS was added on top of the hydrogels. The absorbance of RhoB at *λ* = 555 nm was recorded by an ultraviolet-visible (UV–vis) spectrophotometer (Cary60, UV-Vis spectrophotometer). The fluorescence of Albumin-FITC *λ* = 495 nm was recorded by a fluorescent spectrophotometer (Cary300, spectrophotometer). The tests were conducted three times at RT. The Korsmeyer–Peppas equation was used to investigate the drug release mechanism of these hydrogels (eqn (2)). It is worth mentioning that this equation is restricted to the first 60% of the released drug.2
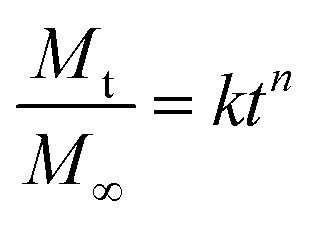
*M*_t_/*M*_∞_ is the cumulative fractional drug release, *k* is a kinetic constant, *t* is the release time and *n* is the diffusional exponent related to the release mechanism. For thin films, when *n* = 0.5, drug release is driven by Fickian diffusion. When 0.5 < *n* < 1, anomalous transport occurs involving both Fickian diffusion and polymer chain relaxation. When *n* value indicates 1, Case II transport is observed.

### 2.9 3D encapsulation of hMSCs in the nanocomposite hydrogels

Bone marrow-derived human mesenchymal stem cells (hMSCs) obtained after informed consent from one donor were grown in a-MEM growth medium (Gibco), supplemented with 10% fetal bovine serum (FBS) and 100 U ml^−1^ penicillin + 100 mg ml^−1^ streptomycin (Gibco). Cells were kept in 175 cm^2^ culture flasks at 37 °C with a controlled atmosphere of 5% CO_2_ and grown until 80% confluence. Cell medium was changed every 2–3 days. Cells were used before passage 6 for all experiments. hMSCs were encapsulated at a final cell density of 4 × 10^6^ cells per ml inside the hydrogels. Briefly, freeze-dried MSN (2 wt%) and PEG powder (10 wt%) were mixed with an hMSC cell suspension (in PBS). Then, DPDS was added to the MSN-PEG–cell mixture in a 1 : 3 v/v ratio and the samples were left to incubate at RT for about 20 min. Then, fresh medium was added on top of the hydrogels to supply cells with nutrients and samples were placed in the incubator (37 °C, 5% CO_2_) for 2 h. Afterwards, medium was removed and hydrogels were left to incubate in fresh culture cell medium for 2 days. To evaluate cellular morphology, cells within the hydrogels were stained for F-actin and nuclei using phalloidin and 4′,6-diamidino-2-phenylindole (DAPI) staining, respectively. At day 2, the hydrogels were fixed with 4% formaldehyde. After fixation, the samples were rinsed with a blocking buffer (3% (w/v) BSA and 0.5% (w/v) Tween in PBS) and permeabilized with 0.25% Triton X-100 in blocking buffer for 2 h. Then, the cells were stained by Alexa Fluor 647-conjugated phalloidin (overnight at 4 °C), followed by DAPI staining (2 h at RT). Confocal microscopy (Leica TCS SP8 CARS) was used to image cells in the hydrogels.

## 3. Results and discussion

### 3.1 Formation and structural characterization of MSN nanocomposite materials

**Fig. 1:**
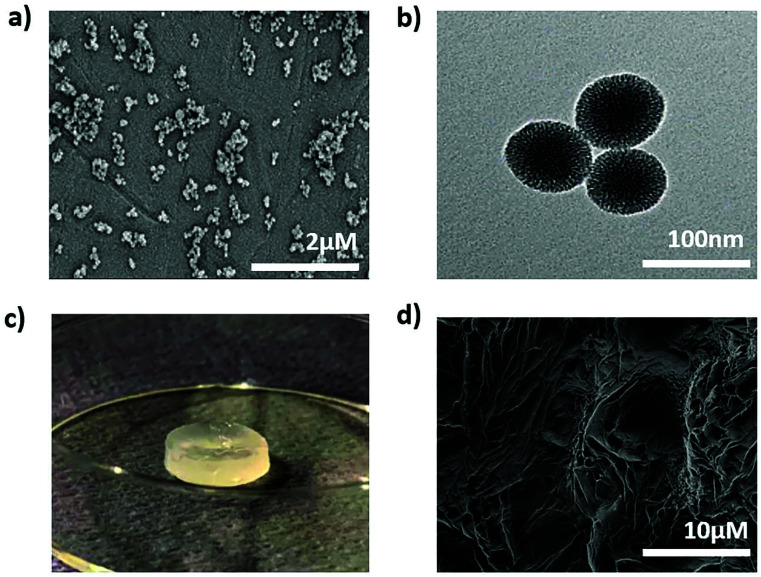
Formation and structural characterization of MSN2-PEG nanocomposite hydrogels. (a) SEM image of MSN-SH, (b) TEM image of the MSN-SH, (c) macroscopic picture of MSN2-PEG, (d) SEM image of freeze-dried MSN2-PEG.

To develop the nanocomposite materials, first thiol surface- and amine core-modified MSNs (MSN-SH) were synthesized, MSNs without surface modification (silanol groups on the surface, MSN-OH) were synthesized as a control. DLS measurements showed that the hydrodynamic diameter of MSN-SH and MSN-OH was 152 nm and 248 nm, respectively, with a narrow size distribution (PDI value was <0.180, Fig. S1†). SEM imaging of MSN-SH confirmed their uniform spherical morphology (Fig. 1a and S1c†). TEM images exhibited that the nanoparticles have a uniform porous structure (Fig. 1b and S1d†). To confirm the successful functionalization of amine groups in the core and thiol groups on the surface, the nanoparticles were labeled with a FITC dye conjugated with NHS group (binds to amines present in the core) and an ATTO dye functionalized with maleimide (binds to the surface thiols), and their fluorescence intensity was measured (Fig. S1b†). As a result, fluorescence intensity for core–shell functionalized MSN-SH was significantly higher than non-functionalized MSN-OH. These results confirm the successful synthesis of MSN-SH and MSN-OH nanoparticles.

To form the nanocomposite hydrogels, 10 wt% of 4-arm-PEG-SH was introduced to 0.1–7 wt% of MSN-SH in the presence of a disulfide-containing initiator (DPDS). Four hydrogel composites were prepared to contain 0.1, 2, 5 or 7 wt% of MSNs, designated as MSN0.1-PEG, MSN2-PEG, MSN5-PEG, and MSN7-PEG, respectively. Hydrogel-like materials that were highly elastic (data not shown) spontaneously formed for all formulations. A whitish color was observed due to the light scattering of the nanoparticles (Fig. 1c), which has been observed previously.^15,31^ A SEM image of MSN2-PEG confirmed a porous network structure (Fig. 1d). The data are shown for MSN2-PEG for simplicity and similar results were observed for the other nanocomposite materials.

### 3.2 Dynamic covalent crosslinking of thiol functionalized MSNs increases the mechanical properties of PEG gels significantly

**Fig. 2:**
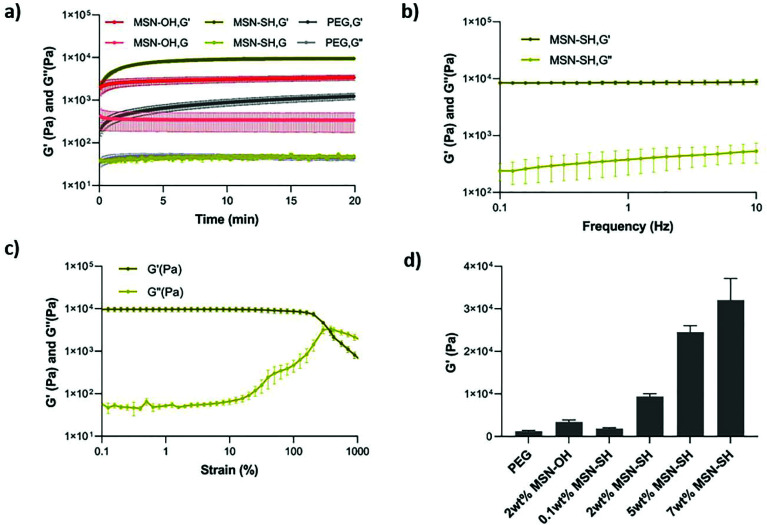
Viscoelastic properties of MSN-PEG nanocomposite materials. (a) Time sweep test results when MSNs act as a crosslinker (MSN-SH), filler (MSN-OH), and without MSNs in the hydrogel network (PEG). All formulations were exposed to a constant frequency of 1 Hz and a strain of 1% during the 20 min incubation at 20 °C. The storage modulus of MSN2-PEG, MSN-OH2-PEG, and PEG hydrogels were 9.4 ± 0.9 kPa, 3.4 ± 0.7 kPa, and 1.3 ± 0.3 kPa, respectively. (b) Frequency sweep test of the MSN2-PEG hydrogel. Measurements were done at a constant strain amplitude of 1% at 20 °C. (c) Strain sweep test of the MSN2-PEG hydrogels. Measurements were done at a constant frequency value of 1 Hz and varying strain amplitudes between 0.1% and 500% at 20 °C. (d) The storage modulus of the MSN-PEG hydrogels with varying MSN wt% compared to MSN-OH2-PEG and PEG hydrogels after they reached equilibrium. Measurements were done at a constant frequency of 1 Hz and strain of 1% at 20 °C. The storage modulus was 1.9 ± 0.3 kPa for MSN0.1-PEG, 9.4 ± 0.9 kPa for MSN2-PEG, 25 ± 2 kPa for MSN5-PEG, and 32 ± 7 kPa for MSN7-PEG hydrogels. All values are presented as mean ± SEM for *n* = 3 per experimental condition.

Next, the viscoelastic properties of the nanocomposite hydrogels were evaluated by oscillatory rheology (Fig. 2 and S2†). At a constant frequency of 1 Hz and strain of 1%, the magnitude of storage modulus was much higher than the loss modulus in all cases, suggesting that all of the formulations exhibit elastic behavior. The gelation occurred within seconds after mixing PEG-SH and MSN-SH solutions in the presence of DPDS and reached an equilibrium in 20 min at 20 °C. The addition of nanoparticles in the network did not significantly influence the gelation time (Fig. 2a and S2†). MSN2-PEG had a storage modulus of 9.4 ± 0.9 kPa, approximately 8-fold higher compared to PEG gels not containing MSNs (1.3 ± 0.3 kPa) and 3-fold higher compared to PEG gels where MSN-OH control particles were incorporated within the PEG gels (3.4 ± 0.7 kPa; Fig. 2a). MSN-OH cannot crosslink with PEG, as they do not contain thiol groups on the surface, thus acting as a filler component in the gel network.

The frequency sweep test was applied to investigate the dynamic nature of the material in the range of angular frequency between 0.1 to 10 Hz. MSN2-PEG hydrogels were stable during a wide frequency range (Fig. 2b). We also determined the critical strain value for disrupting these hydrogels completely and induced a gel–sol transition by applying a strain sweep test (Fig. S2c–e†). When a strain of over 100% was applied, the storage modulus (*G*′) started to decrease significantly, indicating the beginning of the non-linear viscoelastic region. The *G*′ and *G*′′ crossover point defines the critical strain value for the system, which was 381% for MSN2-PEG, demonstrating that these hydrogels can withstand high levels of deformation.

Next, we investigated the effect of different MSN-SH wt% (while keeping the PEG-SH concentration at 10 wt%) on the mechanical properties of the hydrogels. With the lowest MSN-SH content, MSN0.1-PEG exhibited a storage modulus of 1.9 ± 0.3 kPa. With increasing wt% of MSNs in gels, the storage modulus also increased: MSN5-PEG hydrogels had a storage modulus of 25 ± 2 kPa, showing a 20-fold increase compared to pristine PEG-gels (1.3 kPa), and MSN7-PEG hydrogels exhibited a storage modulus of 32 ± 7 kPa, which was an approximately 25-fold increase in G′ compared to PEG hydrogels.

In summary, here we show that by incorporating MSNs with thiol-reactive groups on their surface into a PEG hydrophilic polymeric matrix *via* dynamic covalent bonds, hydrogel-like materials with high mechanical properties could be obtained. Furthermore, by increasing the MSN-SH wt%, the mechanical properties could be modulated over a large range: 1.9 to 32 kPa compared to 1.3 kPa for pristine PEG hydrogels. Our observations that the mechanical properties of PEG gels were also increased when incorporating MSN-OH (which cannot crosslink with PEG) as fillers components in the PEG gels, is in line with previous studies in literature.^15,27,28^ For example, in a study investigating the physical, chemical, and biological properties of photocrosslinked PEG hydrogels containing various concentrations of silica nanospheres as fillers, a significant increase in mechanical properties was observed as a function of silica content.^26^ The reinforcement of hydrogels with silica nanoparticle fillers has been attributed to the physical interaction of polymer adsorption and desorption on the high surface area of silica nanoparticles.^26^ Although the mechanical properties increased when silica nanoparticles were incorporated as filler components, our study shows that the mechanical properties of PEG can be further increased when silica nanoparticles are dynamically crosslinked within the hydrogel network. Similar observations have been reported by Wang *et al.* who prepared silica-grafted double network gels by using silica nanoparticles as macro-crosslinkers to copolymerize in a first network, before polymerization of a second network. The mechanical properties of these gels were compared to silica nanoparticles incorporated in the gels as filler components. Silica-grafted hydrogels showed notably higher compressive strength and elastic modulus compared to 1 wt% silica-filled hydrogels.^29^ In another study, initiator-loaded, hydroxyl-functionalized MSNs (MSN-OH) were crosslinked with macromolecular monomer PEGDA *via in situ* free-radical polymerizations. The mechanical performance significantly improved with an increase of the MSN content; the fracture stress sustained by the PEG hydrogels containing 10 wt% MSNs was about 10 times greater compared to the pure PEGDA hydrogel.^15^

It is worth noting that in our system, a significant increase in storage modulus was observed also at comparatively low MSN content and an overall larger mechanical range could be achieved compared to previous studies. For example, in a study using aminopropyl-functionalized silica nanoparticles and anionic polymers alginate and gellan gum, researchers showed that when silica nanoparticles work as reversible, non-covalent crosslinkers, higher MSN content (>4 wt%) was needed to observe an increase in the storage modulus.^38^ This observation shows that the nature of the crosslinking mechanism, and the polymeric system, are important factors in determining the effect silica nanoparticles will have on the overall mechanical properties of nanocomposites. Indeed, in a study by Fiorini *et al.*, it was shown that incorporating MSNs into the polymeric backbone of a polyamidoamines could also result in a lower *G*′ and *G*′′ compared to the pristine hydrogel.^25^ In conclusion, dynamic disulfide exchange reactions between MSNs surface-functionalized with a high density of thiol groups and 4-arm-PEG-SH resulted in nanocomposites with improved mechanical properties that increased as a function of MSN wt%. To the best of our knowledge, this type of dynamic crosslinking using MSN-SH has not been reported previously.

### 3.3 MSN-PEG nanocomposite hydrogels are self-healing and injectable

**Fig. 3:**
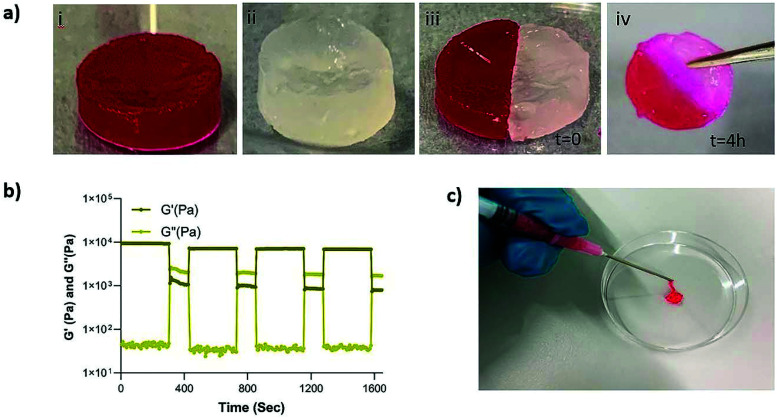
Self-healing and injectable properties of the MSN2-PEG hydrogels. (a) Macroscopic self-healing test. Two cylindrical hydrogels were prepared with RhoB (i) and without a dye (transparent) (ii). After the hydrogels were cut into two pieces by using a scalpel, they were placed together at RT for 4 h. A few drops of initiator solution were added to the interface of the hydrogels to trigger fast reconnection (iii). The two pieces of hydrogels fully self-healed (iv). (b) Rheological recovery test in three stages: (i) time sweep test at 1% strain, (ii) increasing strain from 1% to 500% for 2 min to disrupt the hydrogel network, (iii) recovery at 1% strain for 5 min (*n* = 3). (d) Injectability of the MSN-PEG hydrogels through a 18G syringe needle. RhoB dye was added to the gel for visualization.

Next, we set out to investigate the self-healing and injectability behavior of the nanocomposites. The self-healing behavior of the MSN2-PEG hydrogels was evaluated by a macroscopic self-healing test and rheological recovery tests. Two disk-shaped hydrogels (one of them stained with RhoB) were cut into equal halves by a scalpel and put into direct contact with each other at the side of the cut. After 4 h of incubation at RT, the two differently colored pieces reconnected and could hold their own weight (Fig. 3a). This behavior was attributed to the reformation of broken disulfide bonds at the interface upon bringing together the two hydrogel pieces. Also, a cyclic strain sweep test was performed to confirm self-healing capability of the different formulations of MSN-PEG hydrogels, shifting between 1% (non-deformative) to 500% (highly deformative) of strain amplitude (Fig. 3 and S3†). As illustrated by Fig. 3b, when the 500% strain was applied, the hydrogels exhibited a liquid-like behavior (*G*′ < *G*′′). Upon removal of the high strain, MSN2-PEG recovered spontaneously and showed solid-like behavior (*G*′ > *G*′′). Importantly, rheological measurements confirmed an almost complete recovery of *G*′ within a few seconds after applying and removing the high strain amplitude (500%) (Fig. 3b). We also found that higher MSN concentrations of 7 wt% did not significantly influence the self-healing behavior. However, the recovery of MSN7-PEG was slightly decreased after applying 500% strain (Fig. S3†), likely due to the higher MSN content in the network reducing the mass transfer during the recovery. Moreover, the composite hydrogels were easily extruded through 18-gauge syringe needles, did not block the pinholes (Fig. 3c), and spontaneously reformed as gels after extrusion.

Although not previously observed for MSN-SH nanoparticles, similar self-healing and injectability behavior could be observed for dynamic PEG hydrogels that were prepared by mixing dithiolane-modified PEG and thiol-modified F127 and thiol-functionalized PEG and HAuCl_4_.^11,12^ It was reported that both hydrogel systems, like ours, exhibited a fast recovery after network disruption by a high strain (500%) or by cutting.

### 3.4 Equilibrium swelling degree, degradation and drug release ability of MSN-PEG nanocomposites

**Fig. 4:**
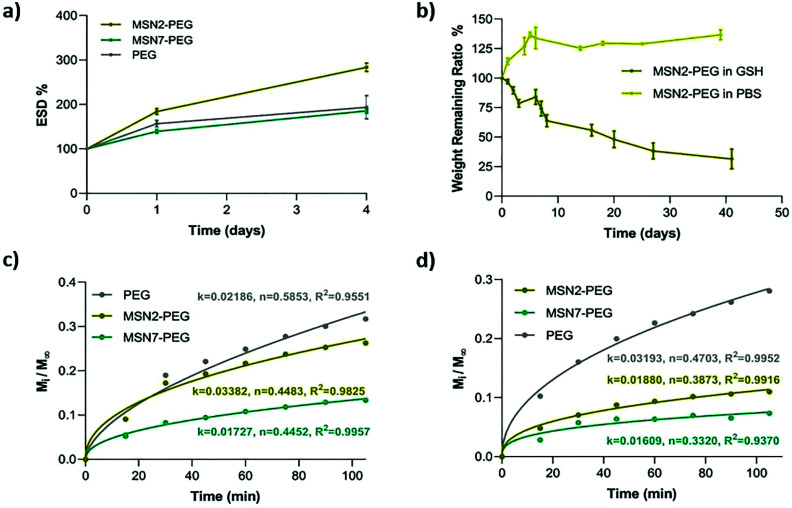
Swelling, degradation, and release behavior of MSN2-PEG, MSN7-PEG and PEG hydrogels. (a) Equilibrium swelling degree of hydrogels in PBS at 37 °C. (b) Degradation of MSN2-PEG hydrogels in 300 μm GSH and in PBS (control) solutions at 37 °C. (c and d) The Korsmeyer–Peppas fitting of the albumin-FITC release data (c) and of the RhoB release data (d).

Equilibrium swelling degree (ESD) is an important gel property that influences the nutrient, growth factor, and oxygen transportation in the network. We tested the ESD of MSN2-PEG, MSN7-PEG, and PEG hydrogels in PBS for 4 days at 37 °C. MSN2-PEG and MSN7-PEG were selected due to their influence on mechanical strength, while PEG was used as a control. The results showed that the lower wt% of MSNs in the network resulted in increased swelling behavior of the hydrogels: MSN2-PEG swelled up to nearly three times its weight (284%) compared to 186% for MSN7-PEG, which was lower than control PEG hydrogels with an ESD value of 193% (Fig. 4a). A similar swelling trend was also reported previously in a different nanocomposite system based on polyamidoamines and MSNs.^25^ It is well known that the swelling degree of hydrogels can be notably affected by their crosslinking density.^39^ Having the highest MSN content in the hydrogel network can lead to denser network formation. Therefore, these nanocomposites have relatively less space to accommodate water molecules, resulting in a lower ESD compared to MSN2-PEG. Similar swelling behavior based on silica content was also reported by Gaharwar *et al.* who showed that the hydration degree was decreased with an increase in silica content in the polymeric matrix.^26^

Next, the degradation behavior of the hydrogels was studied by mass loss studies in physiologically relevant buffer conditions (PBS) or in the presence of glutathione (GSH, 300 μM or 10 mM). The disulfide bonds in the nanocomposite hydrogels are susceptible to degradation in the presence of reducing agents such as GSH. MSN2-PEG lost almost 80% of its mass in the presence of 300 μM GSH over six weeks, compared to no significant mass loss observed in PBS (Fig. 4b). At high GSH concentrations (10 mM), MSN2-PEG completely degraded within 24 h (Fig. S4a†). We also examined the degradation behavior of MSN7-PEG in PBS containing 300 μM GSH. MSN7-PEG exhibited slower degradation than MSN2-PEG; only 8% of the mass was lost during 40 days of incubation period in GSH (Fig. S4b†).

It is worth mentioning that both MSN-PEG hydrogels exhibited relatively slow degradation over time in 300 μM GSH, which is a much higher concentration compared to biological extracellular GSH concentrations (2–20 μM).^40^ In most reported cases, hydrogels formed through disulfide bonds underwent rapid degradation upon exposure to mild GSH concentrations (2–20 μM).^8,13,33^ For example, Choh *et al.* reported that disulfide crosslinked, hyaluronic acid hydrogels degraded completely in varying concentrations of GSH (from 50 μM to 50 mM)-containing media in less than 15 h.^8^ Similarly, Xu *et al.* showed that disulfide crosslinked PLG-*g*-CPA hydrogels degraded completely in 20 μM GSH-containing media in 7 h.^13^

The slower degradation observed in our study may be explained by increased crosslinking density of the MSNs with the polymeric network due to its high surface area. Crosslinking density due to increased polymer concentration has been shown to increase stability against GSH degradation by Yang *et al.*, where 15 wt% PEG-containing hydrogels degraded in 32 days and 3 wt% PEG-containing hydrogels degraded in 2 days in the presence of 10 μM GSH concentration.^14^ In summary, our MSN-PEG hydrogels exhibited notably higher stability in presence of GSH compared to previous studies even at relatively high GSH concentrations. This feature can be advantageous for their application in regeneration of harder and stiffer tissues and organs, since they offer a stable, cell-protective environment for a longer time period.

We further investigated the potential drug delivery properties of nanocomposites by using two model dyes, RhoB and albumin-FITC to mimic small water-soluble molecules and proteins, respectively. Both dyes were readily incorporated into the hydrogels by mixing the dye with the initiator solution. The bulk release profiles for RhoB and albumin-FITC from the hydrogels are shown in Fig. S4c–f.† The highest release was observed in PEG hydrogels with 31% albumin and 28% RhoB; for MSN2-PEG and MSN7-PEG, the release percentages were 11% and 7% for RhoB, and 26% and 13% for albumin after 105 min of incubation, respectively (Fig. S4†). Thus, MSN7-PEG, which has the highest storage modulus, demonstrated the slowest dye release while PEG hydrogels showed the fastest release. Therefore, the release behavior of hydrogels could be modulated depending on MSN content. Interestingly, albumin was released to a similar extent compared to the much smaller molecule RhoB.

To interpret the drug release mechanism for the model dyes, the experimental data were fitted to the Korsmeyer–Peppas equation (Fig. 4c and d).^41–43^ In the equation, the diffusional exponent n shows the mechanism of drug release, *n* ≤ 0.5 characterizing Fickian diffusion-controlled release, 0.5 < *n* < 1, indicating anomalous (non-Fickian) transport of drugs, and *n* = 1 showing the case-II release of drugs. According to Fig. 4c, all the nanocomposite formulations showed Fickian-controlled diffusion behavior (*n* ≤ 0.5) for albumin. Only PEG hydrogels exhibited an anomalous diffusion-controlled profile, likely reflecting the hydrogel's swelling and dissolution behavior, to give rapid release of the cargo. For RhoB release (Fig. 4d), all the hydrogel formulations demonstrated Fickian-controlled diffusion behavior. Thus, MSN-PEG nanocomposites can be used to sustainably release small and large molecules from the network, where the MSN wt% modulates the release kinetics.

### 3.5 MSN-PEG hydrogels allows hMSCs encapsulation

**Fig. 5:**
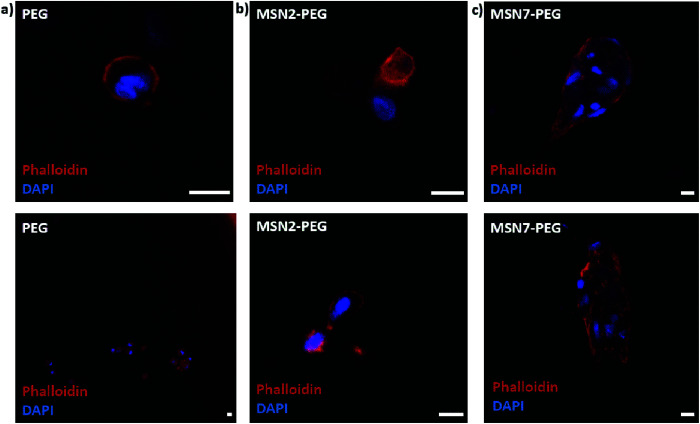
Effect of MSN content on hMSC morphology in a 3D hydrogel environment. (a) hMSCs were encapsulated in pristine PEG (a), MSN2-PEG (b), or MSN7-PEG (c) for 2 days. Samples were stained with DAPI (blue) and phalloidin (red) to visualize the cell nuclei and cytoskeleton, respectively. Scale bars represent 25 μm.

Next, we studied the encapsulation of hMSCs inside MSN-PEG hydrogels with 2 wt% or 7 wt% MSNs, using PEG hydrogel without MSNs as a control (Fig. 5). After 2 days of culture in normal growth medium, the cells were fixed and stained with DAPI and phalloidin to visualize the cell nuclei and F-actin cytoskeletal organization, respectively. Encapsulated hMSCs developed a more stretched morphology when incorporated in MSN-PEG hydrogel with 7 wt% MSNs, compared to a round shape when encapsulated in pristine PEG gels. Moreover, hMSCs encapsulated within MSN2-PEG showed moderate stretching behavior compared to MSN7-PEG. We conclude that MSN-PEG nanocomposites can be used to encapsulate hMSCs and that higher MSN content led to increased stem cell stretching.

Our observations with hMSCs in the MSN-PEG hydrogels are similar to other studies. PEG is a highly hydrophilic, biocompatible polymer. However, it is known to be bioinert resulting in low cell attachment and protein adsorption.^37^ Normally PEG hydrogels need to be engineered to display cell adhesion sites, for example, by using the integrin-binding peptide RGD.^15,27^ Several studies have reported that incorporating MSNs or silica nanoparticles into the hydrogel matrix could also enhance cellular adhesion and spreading.^15,17,44^ For example, Yang *et al.* investigated the effect of MSN content on rat bone marrow stem cell behavior by culturing them on PEGDA-MSN hydrogels.^15^ They reported that the incorporation of MSNs into these hydrogels increased cell adhesion and spreading, as confirmed by the formation of elongated lamellipodia and pseudopodia. They observed more outstretched filopodia-like extensions in higher MSN concentrations (5% and 10%). Gaharwar *et al.* showed that on PEG hydrogel surfaces fibroblasts exhibited a round morphology due to limited cell–matrix interactions, whereas on PEG hydrogels containing silica nanoparticles, a dose-dependent improved cellular adhesion and spreading was observed.^26^

## 4. Conclusions

In this work, we report a new type of nanocomposite hydrogels formed through thiol–disulfide exchange reactions between thiol-functionalized MSNs and thiol-functionalized 4-arm-PEG. We show that when MSNs actively participate in the hydrogel network as dynamic crosslinkers, self-healing biomaterials with high mechanical strength can be obtained. The resulting nanocomposites showed fast gelation, injectability, and rapid self-healing ability while having high mechanical strength. Specifically, the elastic modulus of the nanocomposites could be increased to up to 25-fold compared to pristine PEG hydrogels by changing MSN content, which is an overall larger mechanical range compared to previous studies. Controls using MSNs that were not able to crosslink but acted as filler components, improved mechanical properties only up to 3-fold (at the same wt%) compared to PEG hydrogels, proving the beneficial effect of using MSNs as dynamic crosslinkers on the mechanical properties of self-healing hydrogels. Importantly, high-mechanical strength did not interfere with their self-healing capabilities; fast and a high recovery could be observed for all MSN-PEG composites.

Moreover, these hydrogels were stable under physiological conditions but degraded in a glutathione-containing environment over a period of six weeks. Compared to previous studies, our MSN-PEG hydrogels exhibited notably higher stability in presence of GSH even at relatively high GSH concentrations. This feature can be advantageous for their application in regeneration of harder and stiffer tissues and organs, where regeneration takes place over several weeks to months. Additionally, MSN-PEG could sustainably release two model drugs (RhoB and albumin-FITC), and release kinetics was dependent on the MSN content, demonstrating potential for tenability of drug release. Finally, hMSCs could be encapsulated inside MSN-PEG nanocomposites demonstrating that these materials may also be interesting for use as bioinks to manufacture engineered tissue using 3D printing technology.

In summary, here we show that incorporation of MSNs within hydrogels using dynamic covalent interactions is a promising strategy to overcome current issues associated with low mechanical properties of self-healing hydrogels without affecting their rapid self-healing behavior, widening their applicability in regenerative medicine applications towards mechanically demanding tissues and organs such as cartilage and muscle.

## Conflicts of interest

There are no conflicts to declare.

## Supplementary Material

NR-013-D0NR07406C-s001
