# Supplementary material for: Injectable, self-healing mesoporous silica nanocomposite hydrogels with improved mechanical properties
Source: Nanoscale. 2020 Dec 22;13(2):1144–54. doi: 10.1039/d0nr07406c (PMC8100892; doi:10.1039/d0nr07406c)
Supplement: NR-013-D0NR07406C-s001 [file NR-013-D0NR07406C-s001.pdf]

Electronic Supplementary Material (ESI) for Nanoscale.
This journal is © The Royal Society of Chemistry 2020

Supporting Information

Injectable, self-healing mesoporous silica nanocomposite hydrogels with improved mechanical properties

A. Zengin[1], J.O.P. Castro[1], P.Habibovic[1], and S. van Rijt[1]*

[1]Department of Instructive Biomaterials Engineering (IBE), MERLN Institute, Maastricht University, the Netherlands

*Corresponding Author. E-mail: s.vanrijt@maastrichtuniversity.nl

1. **Structural characterization of MSNs**

a)

	MSN-NH2in-SHout	MSN-OH
Average Size (DLS)	158.5	248
PDI	0.112	0.180

b)

	MSN-NH2in-SHout	MSN-OH
FITC	226623	35
ATTO-633	200623	24

c)

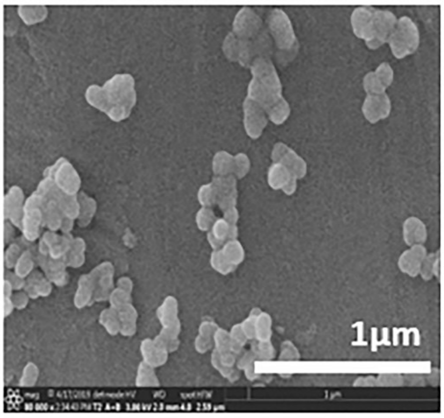

d)

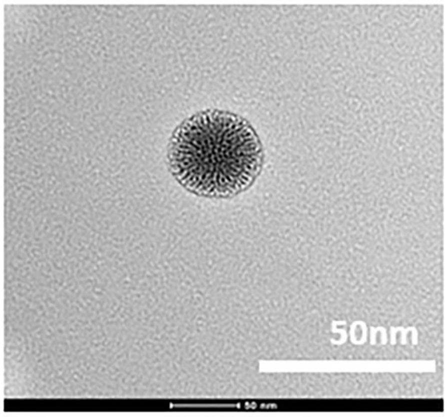

Fig. S1 Structural characterization of MSNs. (a) DLS results of the core-shell functionalized MSNs and non-functionalized MSNs**.** (b) Fluorescence intensity (a.u.) of FITC-NHS and ATTO 633-labeled synthesized MSNs. (c) SEM image and (d) TEM image of MSN-OH.

2. Crosslinking MSNs increased the mechanical properties of PEG gels

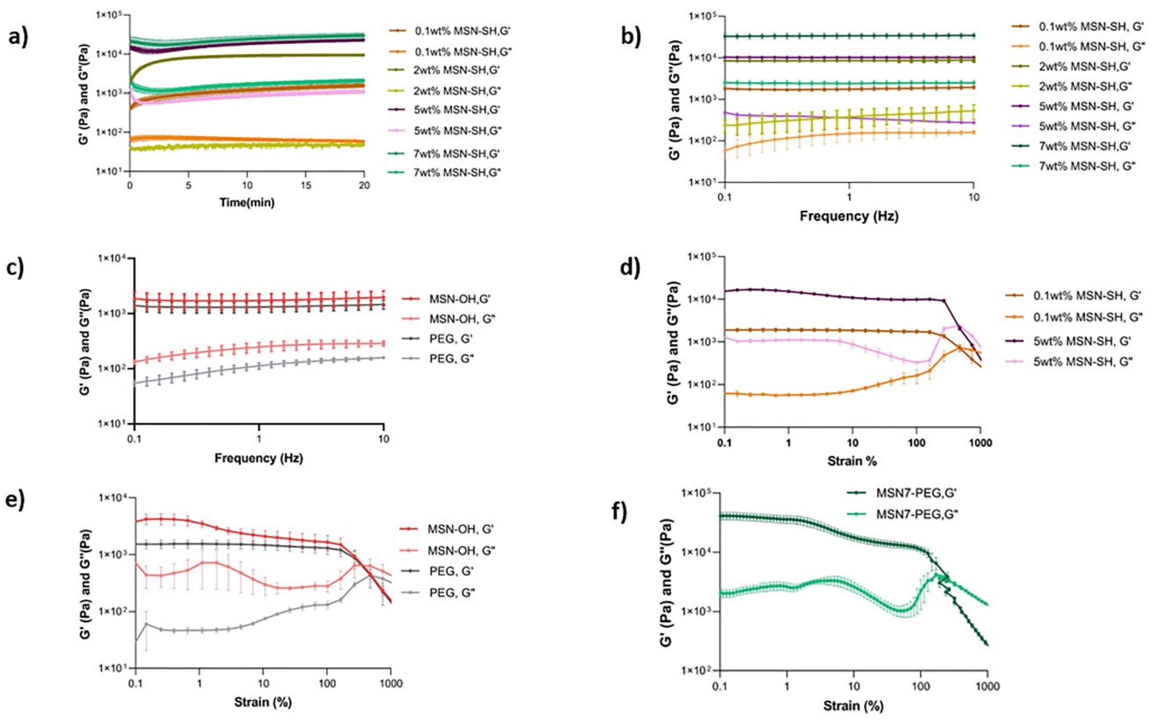

Fig. S2 Rheological analysis of all the hydrogel formulations (MSN0.1-7-PEG, MSN-OH and PEG). (a) Time sweep test at a constant strain of 1% and frequency of 1 Hz for all MSN-PEG formulations. (b) Oscillatory frequency sweep test at a constant strain of 1% for MSN-OH-PEG and PEG gels. (c) Oscillatory amplitude sweep test at a constant frequency of 1 Hz for MSN0.1-PEG and MSN5-PEG gels, in the range of 0.1 to 100%. (d) Oscillatory amplitude sweep test at a constant frequency of 1 Hz for MSN-OH-PEG and PEG gels, in the range of 0.1 to 100%. (e) Oscillatory amplitude sweep test at a constant frequency of 1 Hz for MSN7-PEG gels in the range of 0.1 to 100%. All the measurements were done at 20 °C and they are presented as mean ± SEM for n = 3 per experimental condition.

3. MSN-PEG nanocomposite hydrogels are self-healing.

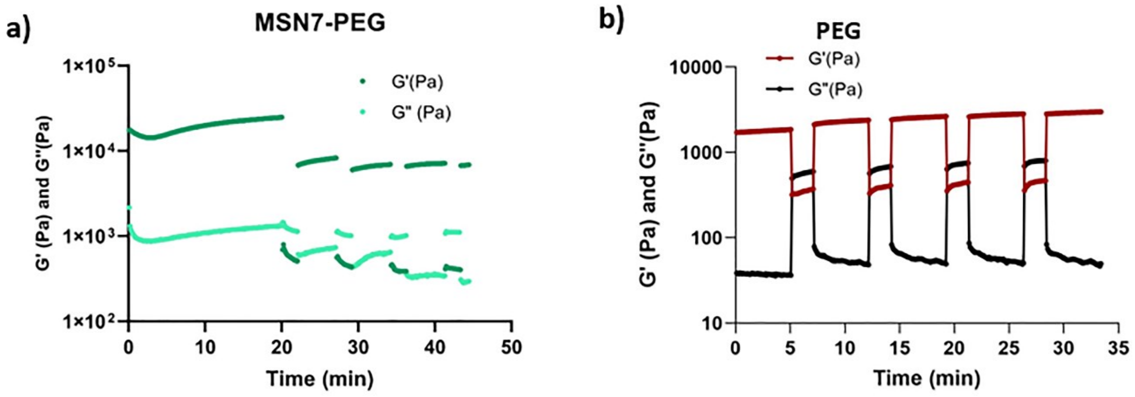

Fig. S3 Cyclic strain sweep test to evaluate the self-healing behavior of the hydrogels. (a) Self-healing tests for MSN7-PEG (a) and pristine PEG (b) hydrogels.

4. Equilibrium swelling degree, degradation and drug release ability of MSN-PEG nanocomposites

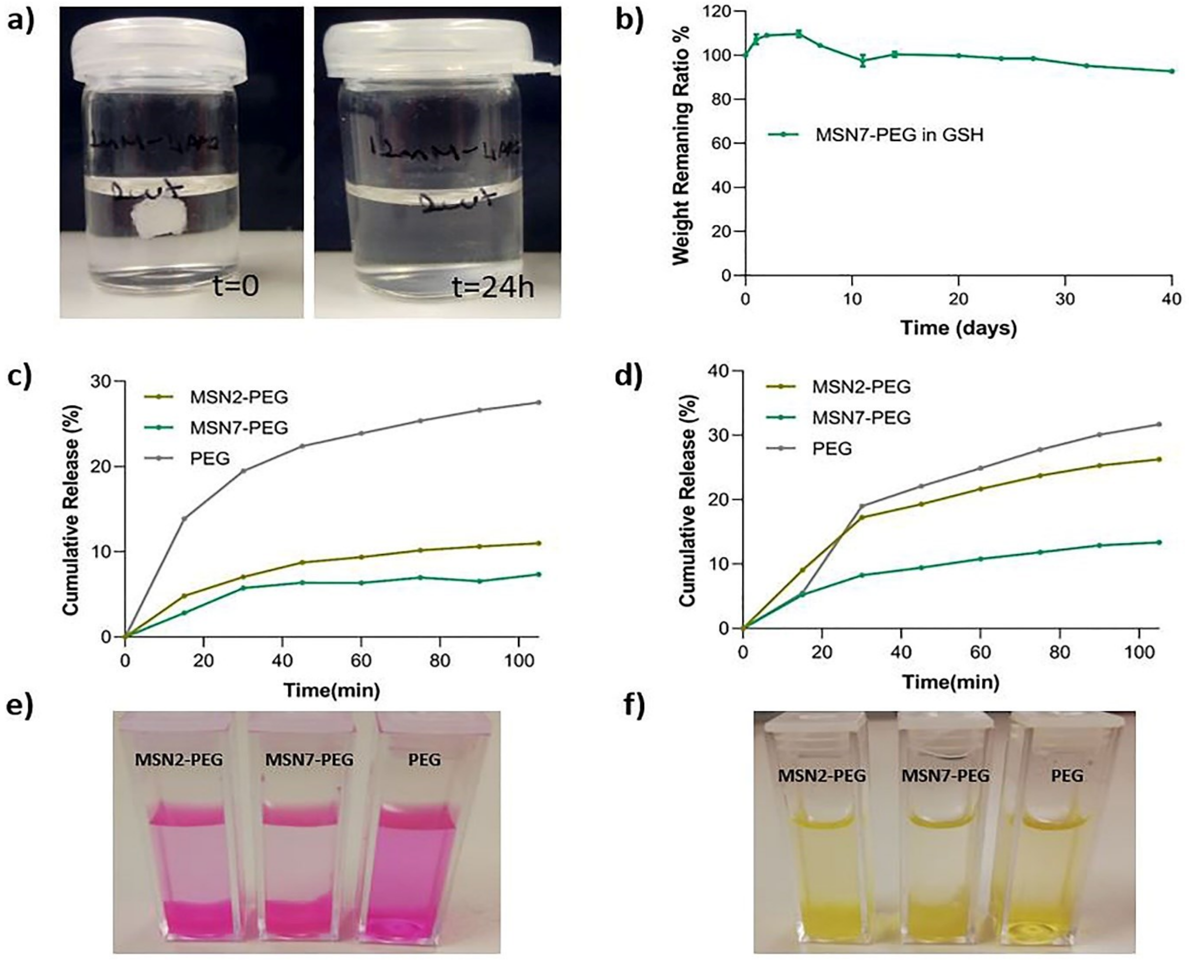

Fig. S4 Degradation and drug release ability of MSN-PEG nanocomposites. (a) MSN2-PEG completely degraded within 24 h in 10 mM glutathione. (b) Degradation of MSN7-PEG hydrogels in 300 μM GSH

at 37 °C. (c) Cumulative release of RhoB release from the hydrogels after 105 min incubation at room temperature. (d) Cumulative release of albumin-FITC release from the hydrogels. (d, e) The representative image of released RhoB (d) and of the released albumin-FITC (e) from MSN2-PEG, MSN7-PEG, and pristine PEG after 105 min.
